# Preliminary Data Related to the Effect of Climacostol Produced by the Freshwater Ciliate *Climacostomum virens* on Human Adenovirus

**DOI:** 10.3390/v12060658

**Published:** 2020-06-18

**Authors:** Marco Verani, Graziano Di Giuseppe, Ileana Federigi, Federico Buonanno, Claudio Ortenzi, Annalaura Carducci

**Affiliations:** 1 Laboratory of Hygiene and Environmental Virology, Department of Biology, University of Pisa, 56127 Pisa, Italy; marco.verani@unipi.it (M.V.); annalaura.carducci@unipi.it (A.C.); 2 Zoology-Anthropology Unit, Department of Biology, University of Pisa, 56126 Pisa, Italy; graziano.di.giuseppe@unipi.it; 3 Laboratory of Protistology and Biology Education, Department of Education, Cultural Heritage, and Tourism (ECHT), University of Macerata, 62100 Macerata, Italy; federico.buonanno@unimc.it (F.B.); claudio.ortenzi@unimc.it (C.O.)

**Keywords:** human adenovirus (HAdV), viral inhibition, climacostol, natural compound

## Abstract

The new epidemiological scenario has so far focused on the environmental circulation of human viral pathogens. Owing to the side effects of chemical disinfectants, there is an increasing need for knowledge on the use of virucidal compounds, especially those of a natural origin. Climacostol is a molecule produced by a freshwater ciliate and it exhibits activity against bacterial and fungal pathogens. We thus also speculated that there might be an effect on viral viability, which has never been tested. To evaluate such activity, we chose human adenovirus (HAdV), which is representative of waterborne viruses. We conducted experiments using HAdV serotype 5, whose titer was determined by infecting HeLa cell cultures. HAdV5 was shown to be sensitive to climacostol at a concentration of 0.0002 mg/mL, with an approximate 3 Log_10_ reduction when the initial titer of HAdV5 was approximately 10^4^ and 10^3^ TCID_50_/mL. These preliminary results could be an important starting point for further research aimed at improving the characterization of climacostol activity under different experimental conditions and against various viruses, including enveloped ones (i.e., the coronavirus). The production of climacostol by a protist living in fresh water also suggests a possible application in the activated sludge of wastewater treatment plants.

## 1. Introduction

The environmental circulation of human viral pathogens is a topic of great interest, particularly today with the occurrence of new epidemiological scenarios [1]. Especially in emergency situations, the need for knowledge on the use of virucidal compounds of a natural origin is even greater, due to the well-known side effects of chemical disinfectants [2]. Several publications underline the role of different natural compounds on viral inactivation and their possible use as alternatives to chemical substances [3,4,5].

**Figure 1:**
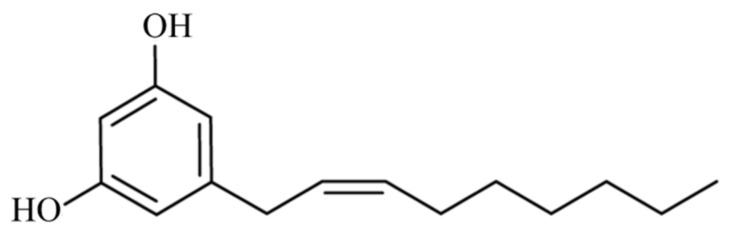
The chemical structure of climacostol.

Eukaryotic microorganisms living in water environments, commonly referred to as protists, are now considered an important source of new bioactive secondary metabolites. Autotrophic species have antimicrobial, antioxidant, anticancer and anti-inflammatory activities [6], and in ciliated protists, many of these compounds appear to be the results of evolutionary selection associated with either attack or defense mechanisms [7]. These substances include climacostol (5-[(2Z)-non-2-en-1-yl]benzene-1,3-diol), produced by the freshwater ciliate, *Climacostomum virens* [7], a colorless toxin essentially used for chemical defense against predators. Climacostol is classified within a large group of natural compounds known as resorcinol lipids, frequently found in plants, fungi and algae. Initially synthesized by Masaki et al. (1999) [8], it was more recently obtained as a pure compound in the natural and most bioactive Z-configuration by a novel and straightforward synthesis [9] (Figure 1).

Studies on climacostol have revealed biological activity on bacterial and fungal pathogens, protozoa, human and rodent cell lines, and isolated mitochondria (see [10] for a review). These findings have indicated that the toxin exerts antimicrobial, cytotoxic, pro-apoptotic and genotoxic activities, and that it also induces dysfunctional autophagy [11].

Based on these various biological proprieties, we also speculated that climacostol could have an effect on virus viability, but to date, no data have been available to confirm this specific characteristic. Considering the habitat of *C. virens*, it would also be interesting to study the effects of climacostol on waterborne viruses, such as the human adenovirus (HAdV). HAdV is a member of the genus *Mastadenovirus* in the Adenoviridae family, and comprises more than 100 different types, several of which in recent years have been identified with molecular techniques [12]. Overall, the types are divided into seven species (A to G) that infect humans, causing a wide range of clinical symptoms, such as upper respiratory tract syndromes (pharyngitis, rhinitis and also pneumonia), gastroenteritis, kerato-conjunctivitis and urinary diseases. HAdVs are also responsible for a large number of asymptomatic infections, which would explain the worldwide distribution of the virus [13].

The main route of transmission is by the inhalation of aerosolized droplets and fecal–oral exposure, since HAdV can be excreted in high levels in stool specimens [12]. Giving the combination of fecal elimination and a relatively high degree of resistance to water disinfection, HAdV has been detected worldwide in various water environments, from wastewaters at various stages of treatment to surface waters (i.e., recreational waters) and tap waters [13]. HAdV has also shown a high frequency and abundance in water environments compared to other enteric, nonenveloped viruses (i.e., norovirus, enterovirus) [14]. HAdV has thus been suggested as an indicator of fecal pollution, and more recently also as an index pathogen for quantitative microbial risk assessment [15].

The aim of our study was a preliminary evaluation of the effect of climacostol on human adenovirus 5 (HAdV5) by in vitro experiments. HAdV5, in fact, is representative of respiratory HAdV, but also, with a waterborne transmission. The obtained data, if significative, could be used as a starting point for a more in-depth research.

## 2. Materials and Methods

### 2.1. Climacostol Preparation

Lyophilized synthetic climacostol (11.6 mg) kindly provided by Prof. Enrico Marcantoni (University of Camerino, Italy) was rehydrated with 70% ethanol, at a concentration of 2 mg/mL, and working solutions of 1 mL were prepared.

### 2.2. Human Adenovirus Cultivation and Quantification

HAdV5 was obtained by American Type Culture cCollection (ATCC VR-5), and it was propagated and assayed on Henrietta Lacks (HeLa) cell line (ATCC CCL-2, batch n. 59681574) as the host cell culture, according to the manufacturer’s instructions. Briefly, a small volume of virus with 0.1 multiplicity of infection (MOI) was absorbed on 25-cm^2^ flasks for 1 h at 37 °C in a humidified 5% CO_2_ atmosphere, rocking every 20 min to redistribute inoculum. After adsorption, growth medium containing Eagle’s minimum essential medium (EMEM) with 2% fetal bovine serum (FBS), 10% L-glutamine and 0.125% gentamycin, was added and the flasks were incubated for 2–3 days. The cytopathic effect (CPE) was revealed by observation on an inverted microscope of rounded, clump and sloughed cells.

Viral quantification was performed by an endpoint dilution assay and working solutions of 1 mL were used. Briefly, the viral titer was measured using 96-well plastic plates by seeding three ten-fold dilutions of each sample onto 8-well lines containing HeLa cell culture. Then, after five days of observation, the highest dilution producing a CPE in 50% of the inoculated cells was determined using the Spearman–Karber formula based on number of wells presenting CPE, and the results were expressed in 50% tissue culture infective dose per milliliter (TCID_50_/mL) [16].

### 2.3. Climacostol Effect on HeLa Cell Line

In order to choose the right concentration for the subsequent tests, cytotoxicity assays were preliminarily performed to evaluate the effect of climacostol on the HeLa cell line. Six serial dilutions of climacostol working solution, from 2 × 10^−1^ to 2 × 10^−6^ mg/mL, were prepared and seeded into 25-cm^2^ flasks with a confluent HeLa cell monolayer. A negative control of HeLa cell without climacostol treatment has been also prepared. After a 24-h incubation period, the cell morphology was observed under the inverted microscope. All six dilutions and the negative control were analyzed in triplicate.

The lowest concentration of climacostol that revealed no effect on HeLa growth and morphology was compared with the concentration of climacostol responsible for 50% cell viability for HeLa cells, defined as 50% cytotoxic concentration (CC_50_). The value of CC_50_ has been obtained from previous experiments using a 3-(4,5-dimethylthiazol-2-yl)-2,5-diphenyltetrazolium bromide (MTT) assay for estimating cell viability, as described by Perrotta et al. (2016) [17].

### 2.4. Climacostol Effect on HAdV5

Three experiments, carried out in triplicate, were designed to test the effect of climacostol concentration coming from Section 2.3 on HAdV5. A total of 1-mL suspension was prepared with 900-µL climacostol (Section 2.3) and 100-µL HAdV5 at three different infectious titers, which were chosen on the basis of the mean viral estimated concentrations found in the water environment, namely 3 × 10^5^, 4 × 10^4^, 3 × 10^3^ TCID_50_/mL [15]. For each assay, a negative control was prepared using 900-µL deionized water and 100-µL HAdV5. To compare our results with studies on water disinfection, the experiments were performed at room temperature (23–25 °C), and the contact time between climacostol and HAdV5 was set at 30 min.

### 2.5. Data Analysis

The CC_50_ values were analyzed and represented using GraphPad Prism (GraphPad Software 7.0, San Diego, CA, USA). The other results were analyzed with Excel for Windows (Microsoft Office Excel 2016, Redmond, Washington, USA) for the calculation and graphical representation of means, standard deviations and viral abatement.

The viral abatement was expressed both as percent reduction and logharitmic (Log_10_) reduction. The percent reduction was calculated using the formula R% = (1 − N_t_/N_0_) × 100, and the Log_10_ reduction was calculated as LR = Log_10_(N_0_/N_t_), where N_0_ is the viral titer at the beginning of each experiment, and N_t_ is the viral titer estimated after the contact time (30 min).

## 3. Results

### 3.1. Results of Climacostol Cell Culture Assays

**Figure 2:**
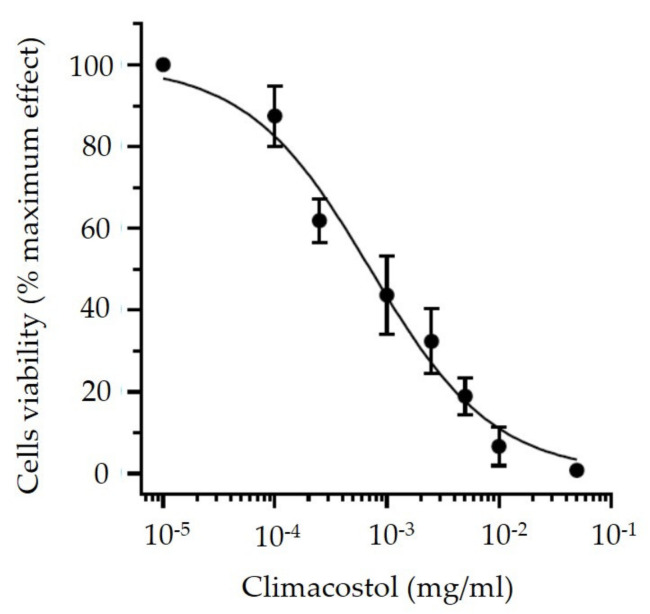
CC_50_ (mg/mL) of climacostol related to HeLa cell lines.

A clear effect on HeLa cells with the alteration of morphology and loss of viability was revealed for the dilutions ranging from 0.2 to 0.002 mg/mL, while the cells remained alive for the other climacostol dilutions. Based on these results, the concentration of 0.0002 mg/mL represented the lowest dilution that did not influence the replication of HeLa cells. This result was in accordance with the CC_50_ value of climacostol (0.0007 mg/mL) based on available data (Figure 2).

### 3.2. Results of Climacostol Effect on HAdV5

**Figure 3:**
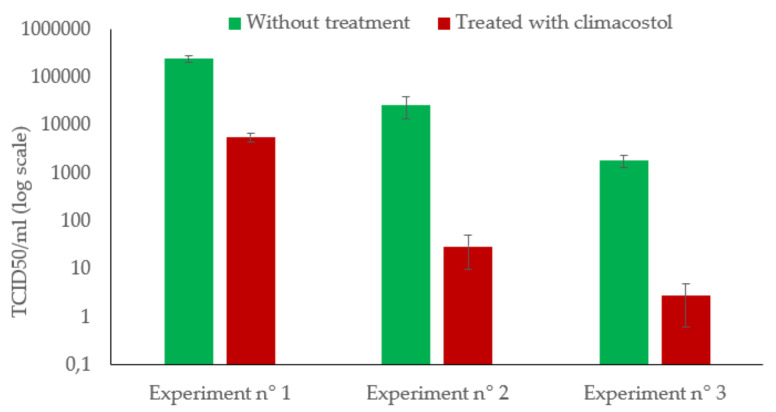
Variation of the HAdV5 titer before and after treatment with climacostol.

For each experiment, the reduction effect on HAdV5 replication was evaluated by comparing the viral titers of the negative control and the suspension treated with climacostol (Figure 3). Under the experimental conditions, our results showed that climacostol was able to reduce HAdV5 infectivity. We observed a 1.62 Log_10_ (97.6%) infectivity decrease when the initial titer of the suspension was 2.36 × 10^5^ TCID_50_/mL, and around a 3 Log_10_ (99.9%) reduction, namely a 2.90 Log_10_ and 2.80 Log_10_ abatement, when the starting HAdV5 titer was 2.58 × 10^4^ and 1.82 × 10^3^ TCID_50_/mL, respectively (Table 1).

## 4. Discussion and Conclusions

In order to compare our results with the literature, we need to consider that most of the HAdV inactivation studies were performed to evaluate the efficiency of chemical disinfectants (i.e., chlorine, chlorine dioxide) or UV irradiation [18], because they are commonly used in water treatments, mainly to disinfect drinking water. For example, Thurston-Enriquez et al. (2003) [19] conducted water experiments to test the chlorine inactivation on HAdV40, and found a 99.9% reduction in buffered demand-free water (0.31 mg/L free chlorine, pH 8.5 °C). These results have been confirmed by other groups [20,21] who performed inactivation assays in similar experimental conditions, also comparing different types of HAdV, including the respiratory types 2 and 5. The viral reduction values found in our study are similar to the results of de Abreu Correa et al. (2012) [22] obtained in their experiments on the abatement of HAdV2 in seawater after 30 min exposure with 2.5 mg/mL free chlorine (approximately 2.7 Log_10_ reduction). Chlorine can produce toxic by-products due to its interaction with organic matter in water, so the use of natural compounds can be an alternative for water disinfection against viral pathogens, as recently highlighted by Garcia et al. (2019) [23]. These authors obtained HAdV abatement in water of 2.5 Log_10_ after 120 min exposure with N-chlorotaurine (NCT) and grape seed extract (GSE) and of 4 Log_10_ after 10 min with bromomamine-T (BAT). Considering the data after 30 min (the contact time adopted in our study), NCT and GSE determined a HAdV abatement of approximately 0.8 Log_10_, while BAT reduced the viral infectivity of 2.5 Log_10_.

However, these data are not comparable with the results of our study, because we tested climacostol inactivation efficiency in a viral suspension and not in a water environment.

In conclusion, to the best of our knowledge, this is the first study demonstrating the effect of climacostol on viral viability, using HAdV5 as a surrogate of human waterborne viruses. Although these findings are very preliminary, they represent a starting point for future research to better understand the molecular mechanism of climacostol action on viral particles. Further investigations are therefore necessary to evaluate the climacostol activity under different conditions, also taking into account the temperature, ionic strength, pH and contact time. Moreover, experiments with natural compounds and chemical substances could be performed in parallel, in order to compare the virucidal action of different types of disinfectant agents.

Nevertheless, these preliminary results on climacostol are interesting for the possible application of this natural compound (and the protists producing it) in water treatments. As an example, wastewater treatment plants could benefit from the presence of ciliated protists producing climacostol in the activated sludge, in order to reduce the viral load in the secondary effluent. It would also be interesting to evaluate its effect on enveloped viruses, such as the coronavirus, whose environmental spread through surfaces and waters should not be underestimated.

## Figures and Tables

**Table 1:** Inhibition of HAdV5 replication by climacostol (fixed at 0.0002 mg/mL) after 30 min of contact time at room temperature.

	HAdV5 Starting Titer (TCID_50_/mL)	HAdV5 Final Titer (TCID_50_/mL)	Log_10_ Reduction	Percentage Reduction
**Experiment 1**	2.36 × 10^5^ ± 0.4 × 10^5^	5.6 × 10^3^ ± 1.2 × 10^3^	1.62	97.6%
**Experiment 2**	2.58 × 10^4^ ± 1.2 × 10^4^	2.96 × 10 ± 1.9 × 10	2.90	99.9%
**Experiment 3**	1.82 × 10^3^ ± 0.5 × 10^3^	2.75 ± 2.13	2.80	99.9%
